# Neuromagnetic activation following active and passive finger movements

**DOI:** 10.1002/brb3.126

**Published:** 2013-02-17

**Authors:** Hideaki Onishi, Kazuhiro Sugawara, Koya Yamashiro, Daisuke Sato, Makoto Suzuki, Hikari Kirimoto, Hiroyuki Tamaki, Hiroatsu Murakami, Shigeki Kameyama

**Affiliations:** 1 Institute for Human Movement and Medical Sciences, Niigata University of Health and WelfareNiigata, Japan; 2 Department of Neurosurgery, Nishi-Niigata Chuo National HospitalNiigata, Japan

**Keywords:** Magnetoencephalography, MEF1, MEG, MRCF, PPC, S2, SEF, SMA

## Abstract

The detailed time courses of cortical activities and source localizations following passive finger movement were studied using whole-head magnetoencephalography (MEG). We recorded motor-related cortical magnetic fields following voluntary movement and somatosensory-evoked magnetic fields following passive movement (PM) in 13 volunteers. The most prominent movement-evoked magnetic field (MEF1) following active movement was obtained approximately 35.3 ± 8.4 msec after movement onset, and the equivalent current dipole (ECD) was estimated to be in the primary motor cortex (Brodmann area 4). Two peaks of MEG response associated with PM were recorded from 30 to 100 msec after movement onset. The earliest component (PM1) peaked at 36.2 ± 8.2 msec, and the second component (PM2) peaked at 86.1 ± 12.1 msec after movement onset. The peak latency and ECD localization of PM1, estimated to be in area 4, were the same as those of the most prominent MEF following active movement. ECDs of PM2 were estimated to be not only in area 4 but also in the supplementary motor area (SMA) and the posterior parietal cortex (PPC) over the hemisphere contralateral to the movement, and in the secondary somatosensory cortex (S2) of both hemispheres. The peak latency of each source activity was obtained at 54–109 msec in SMA, 64–114 msec in PPC, and 84–184 msec in the S2. Our results suggest that the magnetic waveforms at middle latency (50–100 msec) after PM are different from those after active movement and that these waveforms are generated by the activities of several cortical areas, that is, area 4 and SMA, PPC, and S2. In this study, the time courses of the activities in SMA, PPC, and S2 accompanying PM in humans were successfully recorded using MEG with a multiple dipole analysis system.

## Introduction

Several cortical imaging techniques, such as functional magnetic resonance imaging (fMRI), positron emission tomography (PET), electroencephalography (EEG), and magnetoencephalography (MEG), have provided unequivocal evidence of the brain activity in sensorimotor integration (Shibasaki et al. 1980a,b; Kakigi et al. 1995; Mima et al. 1996, 1999b; Weiller et al. 1996; Hari and Imada 1999; Bodegard et al. 2001, 2003; Terumitsu et al. 2009). Compared with fMRI and PET, MEG has excellent temporal resolution and has been used to analyze the temporal aspect of cortical sensorimotor information processing. Cortical activation following application of several stimuli to the peripheral nerves or skin, as well as voluntary movement can be investigated using MEG.

Somatosensory-evoked magnetic fields (SEFs) elicited by electrical stimulation to the peripheral nerves (Hari and Kaukoranta 1985; Nakasato et al. 1996; Kakigi et al. 2000; Inui et al. 2004) or skin (Inui et al. 2003) and by mechanical stimulation, for example, air puff (Karageorgiou et al. 2008), brush (Jousmaki et al. 2007), or mechanical tapping applied to the skin (Hadoush et al. 2010; Onishi et al. 2010), have been investigated in great detail. The major activation induced by electrical or mechanical stimulation to the skin is observed in area 3b of the primary somatosensory cortex (S1), reflecting cutaneous afferents (e.g., Hari and Kaukoranta 1985; Nakasato et al. 1996; Kakigi et al. 2000). Furthermore, many investigators have reported movement-related cortical magnetic fields (MRCFs) following active movement. Neuromagnetic fields over the hemisphere contralateral to the side of the movement change immediately after voluntary movement and are known as movement-evoked magnetic fields (MEFs); these fields are proposed to reflect sensory feedback to the cortex from the periphery. The earliest of these magnetic fields, MEF1, occurs approximately 80–110 msec after the onset of electromyographic (EMG) activity or 20–40 msec after movement onset (Cheyne and Weinberg 1989; Cheyne et al. 1991, 1997, 2006; Kristeva-Feige et al. 1994, 1995, 1996, 1997; Nagamine et al. 1994; Hoshiyama et al. 1997a; Woldag et al. 2003; Oishi et al. 2004; Onishi et al. 2006, 2011).

However, there have been a few studies regarding SEF accompanying passive movement (PM) using MEG systems. Xiang et al. (1997) demonstrated the recording of four SEF components after the onset of passive finger movement. The peak latencies of these components were 20, 46, 70, and 119 msec after movement onset. Several researchers indicated that the large component after PM was of long duration with two peaks from 30 to 100 msec after movement onset (Lange et al. 2001; Alary et al. 2002; Druschky et al. 2003). The equivalent current dipoles (ECDs) of these two components were located in area 3b (Alary et al. 2002), area 4 (Druschky et al. 2003), and areas 3b and 4 (Xiang et al. 1997; Lange et al. 2001). Thus, two components were observed from 30 to 100 msec after PM, and the magnetic waveforms with two peaks following PM were different from the waveforms, with one component following active movement. In contrast, Woldag et al. (2003) reported that the cortical activation patterns and source localizations in active and passive movements were almost identical to those observed in a PET study (Weiller et al. 1996).

Previous PET and fMRI studies have proposed that PM activates an extensive cortical sensorimotor area, for example, the contralateral primary sensorimotor area, supplementary motor area (SMA), posterior parietal cortex (PPC), and bilateral secondary somatosensory areas (S2) (Mima et al. 1996, 1999b; Weiller et al. 1996; Alary et al. 1998; Radovanovic et al. 2002; Albanese et al. 2009). The time courses of activities in these cortical areas, however, have not been clarified because PET and fMRI do not have the temporal resolution of MEG. Furthermore, many MEG studies have not shown evidence of activities in motor-related cortical areas outside the primary sensory and motor areas contralateral movement following PMs (Xiang et al. 1997; Lange et al. 2001; Alary et al. 2002; Woldag et al. 2003).

In this study, we recorded MRCFs following voluntary finger movement and SEFs following passive finger movement in order to examine in detail the differences in cortical activation patterns and source localizations between active and passive movements. We hypothesized that the time course of cortical activities in SMA, PPC, and S2 following PM would be recorded by MEG using a multiple dipole analysis system.

## Methods

### Participants

Thirteen healthy, right-handed volunteers (age, 22–48 years; mean age, 30.8 years; 12 men, 1 woman) participated in this study. All subjects gave their written informed consent. This study was approved by the ethics committee at the Niigata University of Health and Welfare.

### Experimental method

The subjects were seated comfortably inside a magnetically shielded room (Tokin Ltd., Sendai, Japan). All subjects performed the active and passive movement tasks with the right index finger at the metacarpophalangeal (MP) joint. MRCFs elicited by active finger extension and SEFs elicited by passive finger extension and by median nerve stimulation were recorded.

For MEG measurements, the subjects rested their arms comfortably on the armrest of a wooden table, with their hands in full pronation. The right index finger was placed on a small acrylic plate with a light-emitting diode (LED) sensor on the wooden table. The index finger was set at approximately 40º of the MP joint flexion with full extension of the proximal interphalangeal (PIP) joint, and the MP and PIP joints of the third to fifth fingers were kept flexed.

The subjects were instructed to extend their index finger with a brisk movement to reach an adjustable line set up approximately 3 cm above the plate, after completely relaxing the upper limb muscles, at self-paced intervals of approximately 5 sec. The extended position from the active movement was sustained for a moment. When the fingertip was detached from the plate by index finger extension, the LED was cut off, and a trigger signal input averaged the MEG signals online.

For the PM task, an experimenter sat in the magnetically shielded room to the side of the subject. The experimenter passively raised surgical tape (Keeppore25; Nichiban, Tokyo, Japan) wrapped around the subject's index finger with a sharp movement and sustained the index finger in the extended position for a moment, thereby moving the finger with approximately the same motion as that in the active movement task. The interval used for the PM task was approximately 5 sec, which was same as that used for the active movement task. The subjects had several training trials in order to learn to relax their finger and forearm during the PM.

To compare the pattern of brain activity between active and passive movements, the onset of movement, rather than that of EMG activity, triggered the MEG recording. EMG was recorded at the extensor indicis muscle to ensure appropriate execution of active and passive movements. Ag/AgCl disc electrodes were mounted in a bipolar arrangement over the extensor indicis muscle at a distance of 2 cm. The experimenter outside the shielded room confirmed the EMG activity during the PM.

To obtain a reference location of ECDs compared with the locations of magnetic fields elicited by active and passive movements, right median nerve electrical stimulation was applied at the wrist with a monophasic square-wave impulse of 0.2-msec duration at 1.5 Hz. The intensity of electrical stimulation was 1.2 times the motor threshold.

### Preexperiment for confirmation of kinematic data

Before the MEG experiment, we confirmed the speed of active and passive movements, range of motion, and reaction time of the output trigger signal of the LED sensor outside the shielded room. An electrogoniometer (SG65; Biometrics Ltd., Ladysmith, VA) was attached at the MP joint of the right index finger, and the active and passive movement tasks were performed at almost the same frequency (0.2 Hz) as that in the MEG study. EMG was recorded at the extensor indicis muscle and finger flexor muscle to ensure appropriate execution of active and passive movements. Disposable Ag/AgCl surface electrodes (Blue-sensor NF-00; Ambu, Denmark) were mounted in a bipolar arrangement over the muscle at a distance of 2 cm. EMG signals were amplified (DL-140; 4 Assist, Japan), and band-pass filters (5–500 Hz) were used. Continuous data from the LED trigger signal, electrogoniometer signal, and EMGs were digitized at 1000 Hz (PowerLab; AD Instruments, CO). The speed of movement, range of motion, and reaction time of the LED trigger signal after active and passive movements were measured.

### MEG data acquisition

Neuromagnetic signals were recorded using a 306-channel whole-head MEG system (Vectorview; Elekta, Helsinki, Finland). This 306-channel device contains 102 identical triple sensors, each housing two orthogonal planar gradiometers and one magnetometer. This configuration of gradiometers specifically detects the signal just above the source current. Continuous MEG signals were sampled at 1000 Hz using a band-pass filter ranging between 0.03 and 330 Hz.

Before MEG measurements, three anatomical fiducial points (nasion and bilateral preauricular points) and four indicator coils on the scalp were digitized using a three-dimensional (3-D) digitizer (FASTRAK™; Polhemus, Colchester, VT). The fiducial points provided spatial information necessary for the integration of magnetic resonance images (MRI) and MEG data, whereas the indicator coils determined the position of the subject's head in relation to the helmet. T1-weighted MRI was obtained using a 1.5-T system (Signa HD; GE Healthcare, Milwaukee, WI).

### MEG data analysis

The signal space separation (SSS) method, which separates brain-related and external interference signals, was first applied to reduce environmental and biological noise (MaxFilter software 2.2; Elekta, Helsinki, Finland). SSS efficiently separates brain signals from external disturbances based on the fundamental properties of magnetic fields (Taulu et al. 2004; Taulu and Simola 2006).

The data were obtained 1500 msec before and 1000 msec after application of each trigger for MRCFs and SEFs elicited by PM. The averages of approximately 60 epochs for MRCFs and SEFs following PM were obtained separately. SEFs accompanying median nerve stimulation were obtained 50 msec before and 300 msec after stimulation, and 300 epochs were averaged. For analysis of MRCFs and SEFs elicited by PM, the band-pass filter was set from 0.2 to 60 Hz. The data 500 msec before and 500 msec after movement onset were used to analyze MRCFs following active movement and SEFs following PM, and the first 200 msec (−500 to −300 msec) were used for baseline data. To analyze SEFs elicited by median nerve stimulation, the band-pass filter was set from 0.5 to 100 Hz, and the 20-msec period preceding the stimulus was used for the baseline data.

We first calculated the magnitude of the response at each sensor to find the location with the largest response. This was obtained by squaring MEG signals for each of two planar-type gradiometers at a sensor's location, summing the squared signals, and then calculating the root of the sum (Kida et al. 2006, 2007). We used the root sum square (RSS) waveforms to look for a peak channel showing the largest amplitude. Then, the peak amplitude and latency of the prominent response in the RSS waveform were measured at the peak channel to compare MRCFs and SEFs elicited by PM.

As several cortical activities following PM overlapped temporally, we attempted to use multiple source model analysis for the active and passive movements. We used the Brain Electrical Source Analysis (BESA) software package (NeuroScan Inc., Mclean, VA) for the analysis of multiple source locations and time courses of source activities (Inui et al. 2003, 2004; Wang et al. 2004). This method allows spatiotemporal modeling of multiple simultaneous sources over defined intervals. The location and orientation of the dipoles were calculated by an iterative least-squares fit. The goodness-of-fit (GOF) indicated the percentage of the data that could be explained by the model. We used GOF for individual data for a period from 10 to 100 msec after movement onset to determine whether the model was appropriate. GOF (10–100 msec) values >80% were considered to indicate a good model.

First, the best location and orientation of a source for explaining the major magnetic field components was estimated using the one-source model at a point of peak waveform from 10 to 50 msec after movement onset in all subjects. Next, a second source was determined at the next peak amplitude, between 50 and 100 msec, by the distribution of residual magnetic fields. When GOF (10–100 msec) of the residual magnetic fields was <80%, we attempted to find the third source by the distribution of the residual magnetic fields for a period from 10 to 100 msec after movement onset. If the dipole was located outside the sensory and motor cortices in both hemispheres (e.g., below the corpus callosum or around the eye) or GOF (10–100 msec) was <80%, we repeated this procedure until GOF was >80% or four sources were obtained around the sensorimotor area in the hemisphere contralateral to the movement.

The source location was expressed using an MEG head-based coordinate system. The origin was the midpoint between the preauricular points. The *x*-axis indicated the coronal plane with a positive value toward the right preauricular point, the *y*-axis indicated the midsagittal plane with a positive value in the anterior direction, and the *z*-axis indicated the transverse plane preauricular to the *x*–*y* plane with a positive value toward the upper side. The ECD locations were converted into a Talairach-transformed anatomical brain image using BESA and Brain Voyager QX 2.6 (Brain Innovation B.V., Maastricht, Netherlands) and group comparisons were made.

### Statistical analysis

Data are expressed as mean ± SD. Paired *t* tests were used to test for statistical differences in kinematic data between active and passive movements, and in peak latencies between MEF1 and the earliest MEG component after PM (PM1). The statistical significance of source localization at N20m, MEF1, and PM1 was assessed by the Friedman test, and the Wilcoxon rank test was performed for the post-hoc test using *x*, *y*, and *z* coordinates. *P* < 0.016 was considered significant.

## Results

### Kinematic data

**Figure 1:**
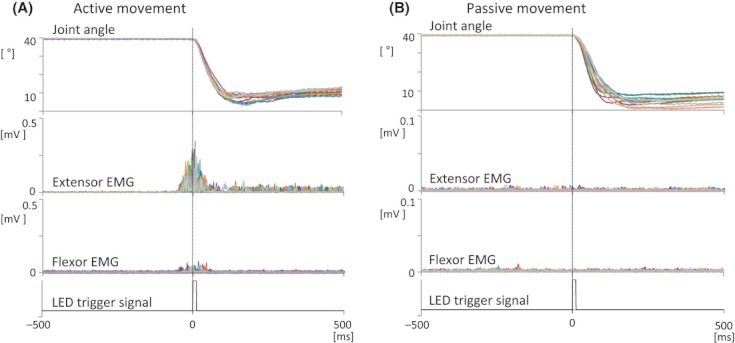
Kinematic data obtained in the preexperiment conducted outside the shielded room from a representative subject. The data recorded from 10 trials are superimposed. The MP joint angle, EMG activity of the extensor indicis and finger flexor muscles, and LED trigger signals 500 msec before and 500 msec after the LED trigger signal accompanying active (A) and passive (B) movements are shown. MP, metacarpophalangeal; EMG, electromyographic; LED, light-emitting diode.

Figure 1 shows the kinematic data obtained during the preexperiment conducted outside the shielded room. Range of motion of the MP joint determined using the electrogoniometer was 26.6 ± 3.3° during active movement, which was not significantly different from the range of motion during PM (27.8 ± 2.6°). The time from movement onset to the maximum extended position was 112.7 ± 16.3 msec for active movement and 120.5 ± 10.5 msec for PM, which were not significantly different. The time lag between the onset of the LED sensor and the onset of deflection of the MP joint observed using the electrogoniometer was <±2.0 msec for both active and passive movements. EMG activities in the extensor indicis muscle occurred 49.5 ± 5.6 msec before the onset of active movement (onset of the LED sensor), and slight activations of the flexor muscle were observed during active movement. No EMG activity was observed in the extensor or flexor muscle during PM.

In the MEG experiment conducted inside the shielded room, EMG activity of the extensor indicis muscle was observed 49.3 ± 6.9 msec before the onset of active movement. No statistical difference was observed in the electromechanical delay from the onset of EMG activity to the onset of movement between the MEG experiment conducted inside the shielded room and the preexperiment conducted outside the shielded room. Furthermore, as in the preexperiment conducted outside the shielded room, no EMG activity was observed in the extensor indicis muscle during PM in the MEG experiment.

### MEG signal amplitude (RSS)

**Figure 2:**
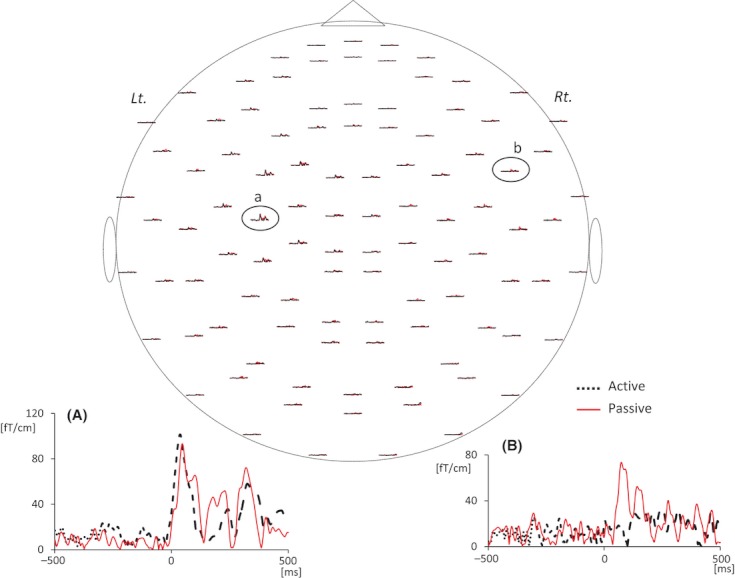
Whole-head distribution of the RSS waveforms from a representative subject following active and passive movements. Enlarged responses from the encircled channels are shown below. Channel (A) is located above the sensorimotor cortex contralateral to the movement, and channel (B) is located above the hemisphere ipsilateral to the movement. The two superimposed lines represent active and passive movements. Lt., left temple; Rt., right temple.

**Figure 3:**
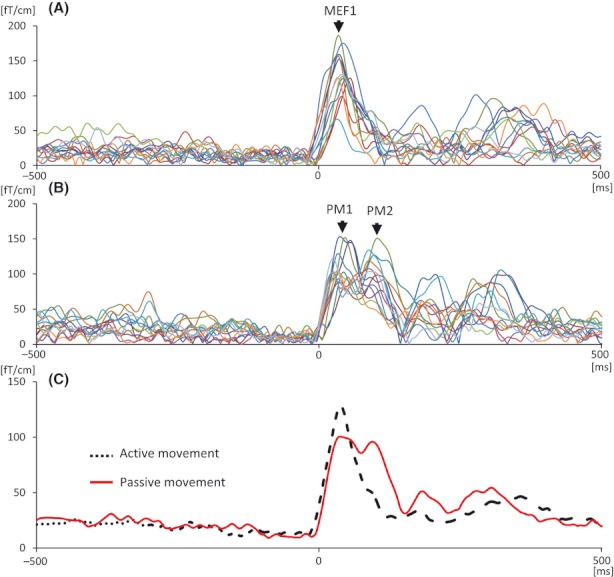
(A) The superimposed RSS waveforms from all subjects at the sensor showing the largest activation following active movement. (B) The superimposed RSS waveforms from all subjects at the sensor showing the largest activation following passive movement. (C) The averaged RSS waveforms of active and passive movements from all subjects.

Figure 2 shows the whole-head distribution of the RSS waveforms from a representative subject 500 msec before and 500 msec after movement onset following active and passive movements, with the enlarged RSS waveforms from two locations during active and passive finger extensions. In all subjects, the largest amplitudes for both active and passive movements were elicited from the same sensor at the sensorimotor area over the hemisphere contralateral to the movement. The small response over the hemisphere ipsilateral to the movement was elicited only by PM and only in some subjects. Figure 3 shows the superimposed RSS waveforms obtained from all subjects at the sensor of the greatest response in each subject following active and passive movements. The large MEF1 response was elicited immediately after the onset of active movement in all subjects (Fig. 3A). In contrast, two peaks in the RSS waveform were clearly elicited immediately after the onset of PM (Fig. 3B) and were referred to as PM1 and PM2, respectively. The averaged RSS waveforms of all subjects following active and passive movements are shown in Figure 3C. Table 1 shows the latencies and amplitudes of the peak responses in all subjects. The peak latency of MEF1 was observed 35.3 ± 8.4 msec after the onset of movement and 84.6 ± 10.0 msec after the onset of EMG activity. The responses following PM over the hemisphere contralateral to the movement peaked at 36.2 ± 8.2 msec in PM1 and 86.1 ± 12.1 msec in PM2 after movement onset. No significant difference was observed in latency between MEF1 and PM1. The peak amplitudes of these components were 138.6 ± 43.4 fT/cm in MEF1, 111.4 ± 31.9 fT/cm in PM1, and 103.3 ± 35.1 fT/cm in PM2. In only six subjects, we clearly identified a small response over the hemisphere ipsilateral to the PM. This response peaked at 115.0 ± 29.9 msec, and the peak amplitude was 89.0 ± 31.0 fT/cm.

**Table 1:** Peak latencies and amplitudes of RSS waveforms at the sensor showing the largest activation after active and passive movements in all subjects

	Active movement		Passive movement					
		
	MEF1		PM1		PM2		Ipsilateral	
				
Subject	Latency (msec)	Amplitude (fT/cm)	Latency (msec)	Amplitude (fT/cm)	Latency (msec)	Amplitude (fT/cm)	Latency (msec)	Amplitude (fT/cm)
1	38	228.4	32	150.9	58	88.2	–	–
2	31	129.0	33	99.7	92	89.1	**–**	–
3	33	185.2	40	179.8	96	155.8	136	145.7
4	26	151.6	45	146.5	95	76.4	71	68.6
5	27	117.1	35	102.2	88	126.5	86	101.5
6	29	157.8	26	121.5	87	103.6	128	78.9
7	33	157.9	38	121.2	83	103.3	124	78.5
8	36	127.5	32	84.1	73	175.8	–	–
9	40	117.3	32	93.3	91	57.6	–	–
10	47	161.5	57	91.3	92	122.1	–	–
11	55	88.9	29	91.4	71	93.7	–	–
12	30	125.8	31	103.9	91	97.8	–	–
13	33	54.1	41	62.8	102	52.9	146	60.5
Average	35.3	138.6	36.2	111.4	86.1	103.3	115.0	89.0
SD	8.4	43.4	8.2	31.9	12.1	35.1	29.9	31.0

MEF1, movement-evoked magnetic field 1; PM, passive movement; SD, standard deviation.

### Source locations and time courses of source activities (BESA analysis)

**Figure 4:**
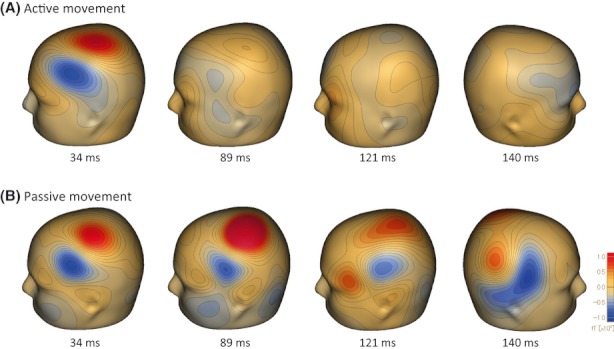
Isocontour maps over the left hemisphere at 34 msec, 89 msec, 121 msec, and over the right hemisphere at 140 msec after active (A) and passive (B) movement in a representative subject. The peak of MEF1 and PM1 after active and passive movements was observed at approximately 34 msec, that of PM2 after passive movement was at 89 msec, that of cS2 after passive movement was at 121 msec, and that of iS2 activity after passive movement was at 140 msec. Red areas indicate magnetic flux exiting the head and blue areas flux entering the head. MEF1, movement-evoked magnetic field 1; PM, passive movement; cS2, contralateral secondary somatosensory cortex; iS2, ipsilateral secondary somatosensory cortex.

Figure 4 shows the isocontour maps over the left hemisphere at 34 msec, 89 msec, 121 msec, and over the right hemisphere at 140 msec after active and passive movements in a representative subject. The field distribution displayed a distinctly different pattern under the active and passive movements. Source activities >80 msec were observed only after the PM.

ECD of MEF1 was located at the sensorimotor area over the hemisphere contralateral to the movement in all subjects. Secondary ECDs after active movement were estimated to be in various areas; for example, at SMA, premotor area, PPC, contralateral secondary somatosensory cortex (cS2), iS2, ipsilateral primary sensory area, and some other areas below the corpus callosum. However, GOF was not >80% after four or five ECDs were estimated, and we could not find a consistent tendency in ECD locations after the first source was estimated following active movement, despite using the multiple source analysis method.

**Figure 5:**
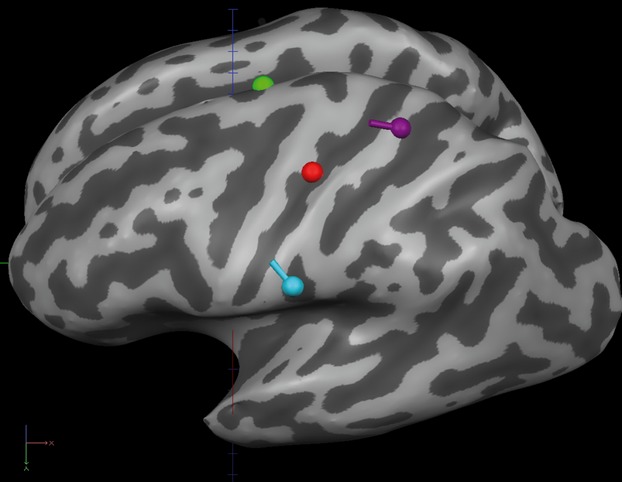
ECDs following passive movement overlapping on the inflated brain of a representative subject. ECDs were estimated at the primary sensorimotor area (red dipole), SMA (green dipole), PPC (purple dipole), and cS2 (blue dipole) in this subject. ECDs, equivalent current dipoles; SMA, supplementary motor area; PPC, posterior parietal cortex; cS2, contralateral secondary somatosensory cortex.

In contrast, we found several ECD locations around the sensory and motor cortices following PM. The first source for the peak of PM1 was estimated to be in the primary sensorimotor area, at almost the same location as that of MEF1 in all subjects. After the first source was estimated, the second, third, fourth, and fifth ECDs were estimated to be at SMA in 12 subjects, PPC in seven subjects, cS2 in seven subjects, and iS2 in seven subjects. Figure 5 presented ECDs following PM overlapping on the subject's inflated brain at a representative subject. ECDs were estimated at primary sensorimotor area, SMA, PPC, and cS2 in this subject.

**Figure 6:**
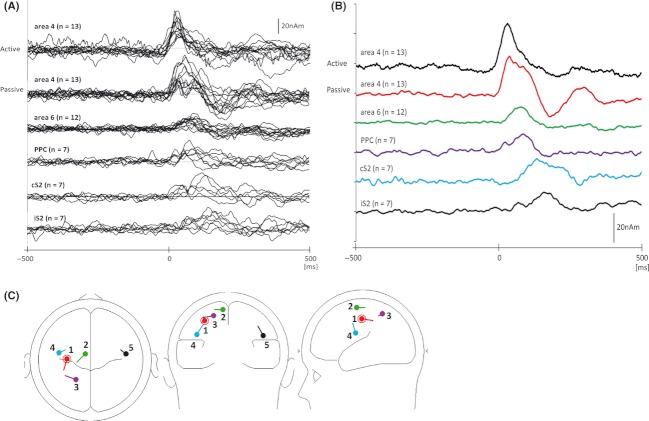
Time course of each source activity and the location of each source using BESA analysis. (A) Time course of each source activity in all subjects. (B) Time course of the averaged source activity of each source. (C) Schematic presentation of locations of all dipoles following passive movement. Area 4 (*n* = 13); SMA (*n* = 12); PPC (*n* = 7); cS2 (*n* = 7); iS2 (*n* = 7). BESA, brain electrical source analysis; SMA, supplementary motor area; PPC, posterior parietal cortex; cS2, contralateral secondary somatosensory cortex; iS2, ipsilateral secondary somatosensory cortex.

The peak latency and moment of each source activity are presented in Table 2. Figure 6 shows the time course of each source activity for all subjects and the average for each source activity following active and passive movements. The peak of the source activities in area 4 was 30.2 ± 10.7 nAm and was observed 33.5 ± 6.3 msec after active movement. The peaks of each source activity were observed 36.0 ± 11.6 msec in area 4, 74.5 ± 16.0 msec in SMA, 89.7 ± 19.7 msec in PPC, 129.4 ± 20.4 msec in cS2, and 128.0 ± 38.4 msec in iS2. The peak activities were 29.2 ± 12.2 nAm in area 4, 14.8 ± 5.1 nAm in SMA, 17.8 ± 5.9 nAm in PPC, 19.7 ± 4.8 nAm in cS2, and 19.7 ± 3.6 nAm in iS2.

**Table 2:** Peak latencies and moments of each source in all subjects following active and passive movements using BESA analysis

	Active movement		Passive movement										
		
	MEF1 (area 4, *n* = 13)		1st source (area 4, *n* = 13)		2nd–5th sources (SMA, *n* = 12)		(PPC, *n* = 7)		(cS2, *n* = 7)		(iS2, *n* = 7)		
							
Subject	Latency (msec)	Moment (nAm)	Latency (msec)	Moment (nAm)	Latency (msec)	Moment (nAm)	Latency (msec)	Moment (nAm)	Latency (msec)	Moment (nAm)	Latency (msec)	Moment (nAm)	GOF (10–100 msec) (%)
1	28	33.4	32	37.8	77	17.2	114	8.9	–	–	–	–	86
2	29	21.7	35	18.8	61	14.4	104	16.2	–	–	184	19.0	82
3	31	46.6	42	32.3	82	29.2	–	–	116	18.4	98	20.1	84
4	31	24.8	38	21.6	99	9.4	–	–	150	12.1	84	16.1	81
5	30	21.1	34	16.7	60	14.5	105	19.7	–	–	93	14.6	80
6	28	33.8	21	27.3	96	12.1	84	17.2	148	26.1	151	19.9	80
7	30	17.8	35	30.0	54	10.4	–	–	–	–	161	25.1	84
8	39	18.4	58	37.7	57	13.6	–	–	133	21.6	–	–	82
9	41	35.9	26	13.6	76	13.3	92	17.7	145	23.3	–	–	80
10	40	46.9	57	52.2	74	16.6	–	–	95	15.4	–	–	82
11	48	17.0	41	49.3	–	–	64	28.6	–	–	–	–	85
12	29	38.7	22	23.2	63	14.1	65	16.0	–	–	–	–	82
13	32	36.9	27	19.4	95	12.7	–	–	119	21.2	125	22.9	80
Average	33.5	30.3	36.0	29.2	74.5	14.8	89.7	17.8	129.4	19.7	128.0	19.7	82.2
SD	6.3	10.7	11.6	12.2	16.0	5.1	19.7	5.9	20.4	4.8	38.3	3.6	2.0

BESA, brain electrical source analysis; MEF1, movement-evoked magnetic field 1; SMA, supplementary motor area; PPC, posterior parietal cortex; cS2, contralateral secondary somatosensory cortex; iS2, ipsilateral secondary somatosensory cortex; GOF, goodness-of-fit; SD, standard deviation.

Talairach coordinates for the estimated sources are summarized in Table 3. ECDs of MEF1 and PM1 were located at the sensorimotor area over the hemisphere contralateral to the movement, and these ECDs were significantly medial (*P* < 0.01), slightly anterior, and significantly superior (*P* < 0.01) to that at N20m. No significant differences in locations were observed between MEF1 and PM1 in the medial–lateral, anterior–posterior, and superior–inferior directions. The other ECDs obtained following PM were estimated to be located definitely medial, slightly anterior, and superior to those at N20m (SMA, *n* = 12); medial, definitely posterior, and superior to those at N20m (PPC *n* = 7); and at S2 over the hemispheres contralateral (*n* = 7) and ipsilateral (*n* = 7) to the movement.

**Table 3:** Talairach coordinates of the sources estimated using BESA analysis

	*X*	*Y*	*Z*
Median nerve stimulation			
N20m (*n* = 13, area 3b)	−44.3 ± 3.7	−16.7 ± 4.7	47.5 ± 3.8
Active movement			
MEF1 (*n* = 13, area 4)	−37.4 ± 4.0	−13.6 ± 5.9	51.4 ± 7.5
Passive movement			
Source 1 (*n* = 13, area 4)	−39.6 ± 3.4	−14.1 ± 4.4	49.7 ± 3.5
Source 2 (*n* = 12, SMA)	−11.1 ± 4.7	−13.0 ± 5.2	65.9 ± 4.4
Source 3 (*n* = 7, PPC)	−24.6 ± 4.7	−42.0 ± 5.4	56.4 ± 5.4
Source 4 (*n* = 7, cS2)	−49.6 ± 4.7	−14.1 ± 5.0	25.0 ± 4.1
Source 5 (*n* = 7, iS2)	50.4 ± 3.1	−9.7 ± 2.7	27.8 ± 2.9

Data are represented as (mean ± SD, mm).

BESA, brain electrical source analysis; MEF1, movement-evoked magnetic field 1; SMA, supplementary motor area; PPC, posterior parietal cortex; cS2, contralateral secondary somatosensory cortex; iS2, ipsilateral secondary somatosensory cortex; SD, standard deviation.

## Discussion

This study examined detailed neuromagnetic activation following active and passive finger movements. The most prominent magnetic field after active movement (MEF1) was obtained at approximately 35.3 ± 8.4 msec, and the source was located in area 4. Two peaks of MEG response associated with passive finger movement were recorded from 30 to 100 msec after movement onset. The earliest component (PM1) peaked 36.2 ± 8.2 msec after PM, and the peak latency and source location at PM1 were the same as those at MEF1. The second peak (PM2) occurred 86.1 ± 12.1 msec after PM. The sources of PM2 were estimated to be at SMA and PPC over the hemisphere contralateral to the movement.

MEF1 was successfully obtained 35.3 ± 8.4 msec after the onset of finger movement or 84.6 ± 10.0 msec after the onset of EMG activity. Neuromagnetic fields over the hemisphere contralateral to the side of the movement change immediately after voluntary movements, and are referred to as MEF1. These fields are proposed to reflect sensory feedback to the cortex from the periphery, and the peak amplitude of MEF1 occurs 20–40 msec after the onset of movement or 80–110 msec after the onset of EMG activity (Cheyne and Weinberg 1989; Cheyne et al. 1991, 1997, 2006; Kristeva-Feige et al. 1994, 1995, 1996, 1997; Nagamine et al. 1994; Hoshiyama et al. 1997a; Woldag et al. 2003; Onishi et al. 2006, 2011).

ECD of MEF1 was located significantly medial and superior to that at N20m and was not significantly anterior to that at N20m. N20m is accepted as the tangential source in area 3b. Many researchers have reported that the source of MEF1 should be located in the S1, that is, area 3a, which is known to receive predominant input from proprioceptive receptors activated during movement (Cheyne and Weinberg 1989). However, the ECD depths of MEF1 indicate that MEF1 responses do not originate from area 3a, which is located deeper than area 3b. Additionally, area 3a is situated at the bottom of the central sulcus, and the orientation of ECDs generated in 3a is primarily radial toward the brain surface. As radial vectors do not produce an external magnetic field, MEG should be largely blind to generating sources in area 3a (Hari and Forss 1999). Therefore, activities in area 3a may not be recorded even if these areas are activated immediately after movement. On the other hand, it has been reported that ECD of MEF1 located in the precentral area, regardless of MEF1 responses, is the result of afferent feedback from muscles (Woldag et al. 2003; Onishi et al. 2011). It is well known that the muscle afferents project to areas 3a and 2 (Jones 1983). However, several investigators, using electrocorticography in humans (Goldring and Ratcheson 1972; Papakostopoulos et al. 1974; Cooper et al. 1975; Lee et al. 1986) or microelectrodes in monkeys or baboons (Rosen and Asanuma 1972; Wiesendanger 1973; Lucier et al. 1975; Lemon 1979, 1981; Lemon and van der Burg 1979; Fetz et al. 1980) have proposed that the muscle afferents project to the precentral area. Kawamura et al. (1996) reported that ECD of the second peak elicited by median nerve stimulation was medial and superior to that at N20m, on the anterior wall of the central sulcus, “area 4”. The findings of our study and the above-mentioned studies suggest that the MEF1 response might be originating from area 4.

We found two peaks of MEG response associated with passive finger movement from 30–100 msec after movement onset. The peak latency and ECD location of earliest component (PM1) following PM were not significantly different from those of MEF1 following active movement. An fMRI study showed that the activity in area 4 accompanying PM was the same as that accompanying active movement (Terumitsu et al. 2009). As mentioned above, it has been reported that neurons in area 4 receive muscle afferent inputs (e.g., Goldring and Ratcheson 1972). Subdural recording has shown that PM can elicit an initial response at 34 msec in the precentral area (Papakostopoulos et al. 1974; Lee et al. 1986). If a muscle is passively stretched, the afferent input from muscle spindles projects to that area of the cortex that excites cells for contracting the same muscle (e.g., Rosen and Asanuma 1972). Desmedt and Ozaki (1991) reported somatosensory-evoked potentials (SEPs) following PM, and they concluded that the recorded positive response with a mean peak latency of 33 msec at the contralateral precentral site was primarily generated in area 4. Mima et al. (1996) reported SEPs following PM using a unique technique. The evoked responses persisted in spite of the abolition of cutaneous and joint afferents of the finger caused by ischemic anesthesia, but they were lost with ischemic anesthesia of the forearm. Accordingly, they concluded that the cortical-evoked responses following PM reflected forearm muscle afferent inputs. It is thought that PM1 obtained 36 msec after PM in our study reflects muscle afferent inputs accompanying muscle stretching and is primarily generated in area 4, same as that observed in case of MEF1.

After estimating the best dipole for explaining the major magnetic component of PM1, some sources were identified by the distribution of the residual magnetic fields and located at SMA (*n* = 12) and/or PPC (*n* = 7). Time courses of the source activities peaked at 54–109 msec in SMA and 64–114 msec in PPC. In addition, the time course of source activity in area 4 obtained at the peak of PM1 prolonged the activity for this period. The two peaks of magnetic response following PM agree with those observed in previous reports (e.g., Xiang et al. 1997). However, the source locations of PM2 at SMA and PCC over the hemisphere contralateral to the movement are in disagreement with those observed in the previous reports, which estimated that the source 70–100 msec after the onset of PM was located in area 4/3b (Xiang et al. 1997; Lange et al. 2001), area 4 (Druschky et al. 2003), and cS2 (Alary et al. 2002). Because these studies used a single dipole method to estimate the source locations, it may have been difficult to detect the activities of SMA and PPC for consecutive activities in area 4.

SMA, traditionally defined as a motor area, is involved in sequencing multiple movements over time, and neurons in SMA are active in relation to a particular order of forthcoming movements guided by memory (e.g., Tanji 1994). However, SMA, the primary motor area, and the primary somatosensory area are activated with PM without muscle contraction (Weiller et al. 1996; Radovanovic et al. 2002). Reddy et al. (2001), using fMRI, reported SMA activation by PM and the total absence of SMA activation during PMs performed by patients with severe distal sensory neuropathy. They concluded that this cortical activation in SMA after PM was dependent on sensory feedback and was unlikely to be due to mental imagery alone. There have been several electrophysiological studies concerning SMA activity following somatosensory stimulation (Reddy et al. 2001). Human studies using subdural electrodes placed over SMA revealed middle latency (50–100 msec)-evoked potentials following median nerve stimulation (Allison et al. 1991; Barba et al. 2005). In addition, using EEG, Tarkka and Hallett (1991a,b) reported that SMA activity peaked approximately 50–100 msec following PM. Somatosensory signals have access to SMA, and the neurons in SMA are activated at latencies that are only slightly longer than latencies at which neurons in area 4 are activated (Wiesendanger 1986). Thus, our results indicating SMA activity associated with PM are in agreement with those of previous studies using PET and fMRI (Weiller et al. 1996; Reddy et al. 2001; Radovanovic et al. 2002). Furthermore, our time course of SMA activity was similar to that elicited by median nerve stimulation and PM using EEG and electrocorticography.

We located the source of activity in the posterior wall of the postcentral fissure 64–114 msec following PM, and this ECD location was 23.8 mm posterior, 19.3 mm medial, and 9.0 mm superior to the source estimated at N20m. Using BESA analysis, Hoshiyama et al. (1997b) reported that the ECD location of PPC was 24 mm posterior, 19 mm medial, and 26 mm superior to the S1 hand area (Hoshiyama et al. 1997b). Areas 5 and 7 in the posterior wall of the postcentral fissure are considered to be at a higher level than S1 in the processing of somatic information (Duffy and Burchfiel 1971; Sakata et al. 1973; MacKay et al. 1978). Prevosto et al. (2011) identified direct and polysynaptic somatosensory pathways from areas 2 and 3a to PPC, and they found that PPC receives disynaptic inputs from dorsal column nuclei as directly as other somatosensory areas (Prevosto et al. 2011). EEG (Arezzo et al. 1981), PET (Radovanovic et al. 2002), and fMRI (Albanese et al. 2009) studies have also reported that neurons in areas 5 and 7 are activated by PMs. The PPC is most active 70–110 msec after median nerve stimulation (Forss et al. 1994; Mauguiere et al. 1997). In this present study, we have confirmed the activities in PPC and the time course of PPC activity with regard to passive finger movement using MEG.

We have also elucidated the activities of S2 areas following PM over the hemispheres contralateral (*n* = 7) and/or ipsilateral (*n* = 7) to the movement, with these activities peaking approximately 120 msec after the onset of PM. There have been many MEG studies of S2 activities following electrical stimulation (Forss et al. 1994; Mima et al. 1998; Hari and Forss 1999), mechanical stimulation (Hoechstetter et al. 2000, 2001; Onishi et al. 2010), and PM (Xiang et al. 1997; Alary et al. 2002). MEG responses from S2 were bilateral and peaked at 80–150 msec (Forss and Jousmaki 1998). Our results of bilateral S2 responses agree with those of previous reports.

We could not observe MEF with a latency of >150 msec (MEF2) in this study, although MEF2 has been recorded 150–200 msec after the onset of active movement in previous studies (Nagamine et al. 1994; Hoshiyama et al. 1997a; Kristeva-Feige et al. 1997; Cheyne et al. 2006). In addition, we have shown no evidence of activities in SMA and S2 after voluntary movements, although many researchers have reported that active movement is associated with activation of SMA and bilateral S2 areas using fMRI or PET (Rao et al. 1993; Weiller et al. 1996; Mima et al. 1999a). Here, the participants were instructed to maintain the MP joint at the extension position for a moment. As a result, muscle activity continued for >500 msec after movement onset. Consequently, neurons in area 4 remained active during this time to hold the muscle contraction. We previously confirmed that MEF2 amplitude decreases with increased muscle activity (unpublished data). One possibility for our failure to observe MEF2 and SMA and/or S2 activities may be the masking effect by the high activity in area 4. Another possibility may be that interference by voluntary movement such as somatosensory gating effect induces MEF2 diminishment and the PPC and S2 activities following active movement.

PPC and S2 responses were not obtained by median nerve stimulation in this study, although there have been some MEG studies of PPC and S2 responses following median nerve stimulation as mentioned above (e.g., Forss et al. 1994; Mauguiere et al. 1997). The interstimulus interval (ISI) of electrical stimulation was set at >1 sec in these previous studies. Our main focus in this study was to investigate the differences in cortical activation patterns and source localizations between active and passive movements. Therefore, we used the median nerve stimulation to reveal the location of area 3b in the S1. To reduce the total experiment time for the participants, we used the stimulus rate of 1.5 Hz to record the most popular SEF response “N20m” as the reference of ECD location. Wikstrom et al. (1996) reported that the MEG response from PPC and S2 were seen only with ISI of ≥1 sec, beginning strongest at the 5-sec ISI. Therefore, it was considered that the absence of PPC and/or S2 activities following median nerve stimulation might be observed in this study. Further investigations are required for gaining more insight into the PPC and S2 responses following median nerve stimulation and PM.
